# Intimate partner violence and physical and mental health among women utilizing community health services in Gujarat, India

**DOI:** 10.1186/1472-6874-14-127

**Published:** 2014-10-16

**Authors:** Akiko Kamimura, Vikas Ganta, Kyl Myers, Tomi Thomas

**Affiliations:** Department of Sociology, University of Utah, 380 S 1530 E, Salt Lake City, Utah 84112 USA; The Catholic Health Association of India, Secunderabad, Andhra Pradesh India

**Keywords:** Intimate partner violence, Physical and mental health, Women’s health, Community health services, India

## Abstract

**Background:**

Intimate partner violence (IPV) is a significant public health threat which causes injury and acute and chronic physical and mental health problems. In India, a high percentage of women experience IPV. The purposes of this study include 1) to describe the lifetime prevalence of IPV, and 2) to examine the association between IPV and physical and mental health well-being, among women utilizing community health services for the economically disadvantaged in India.

**Methods:**

Women utilizing community health services (N = 219) aged between 18 and 62 years completed a self-administered survey in Gujarat, India. Standardized instruments were used to measure perceived physical and mental health well-being. In addition, participants were asked about their lifetime experience with IPV, and socio-demographic questions. Analysis was restricted to the ever-married participants who completed the questions on IPV (N = 167).

**Results:**

Participants with a lifetime history of IPV were more likely to have reported poorer physical and mental health compared to those without a lifetime history of IPV. More than half of the participants with an IPV history experienced multiple types of IPV (physical, sexual and/or emotional IPV). While being in the highest caste was a significant positive factor associated with better health, caste and other socio-demographic factors were not associated with IPV.

**Conclusions:**

Women in India face risk of IPV. Yet those experiencing IPV do not seek help or rely on informal help sources. Community health organizations may take a role in IPV prevention and intervention. Diversity of intervention options would be important to encourage more women with IPV experience to seek help.

## Background

Intimate partner violence (IPV) is a significant public health threat which causes injury and acute and chronic physical and mental health problems [1–3]. IPV comprises of physical, sexual and psychological violence committed by a current or former intimate partner including a spouse or a dating partner [4]. According to a World Health Organization (WHO) survey of ten countries, the lifetime prevalence of physical IPV varies from 15% to 71% [5].

Previous studies examined physical and mental health consequences of IPV. Physical health problems resulting from IPV include physical symptoms (e.g. irritable bowel disease or fibromyalgia) [1–3, 6–9]. Depression and post-traumatic stress disorders are common mental health consequences of IPV [3, 10]. IPV is also one of the major risk factors of suicide attempts [11]. Because women do not always seek health care immediately following an IPV incident [12], it is often difficult to identify which type of IPV affects what specific health problems especially if a woman experiences multiple types of IPV. But in general, injuries are the major health outcomes associated with immediate healthcare utilization [12, 13]. The impact of each type of IPV or multiple types of IPV on health has been examined but the results of previous studies are not consistent. The results of a study in South Africa indicated that women who experienced emotional abuse only had less depressive symptoms and psychological distress compared to women who experienced physical and/or sexual abuse and emotional abuse [14]. Another study conducted in Japan suggests that there was no significant difference in physical and mental health outcomes between emotional abuse only and all types of IPV [15]. The impact of emotional IPV on health is especially under-studied [15]. Among women who had experienced IPV, those who had more social support reported better physical and mental health than those who had less social support [16].

In India, more than one third of women experience physical or sexual violence some time in their lifetime [17]. The lifetime prevalence of IPV was 37.9% based on the 2005–2006 India National Family Health Survey 3 [18]. The prevalence of IPV was approximately 30% to 40% at orthopedic trauma hospitals [19]. The percentage of women who had experienced IPV is high among outpatient psychiatric patients, over 55%, and the majority of the women who had experienced IPV reported depression [20].

Risk factors of IPV in India include low household income, low education levels, low caste, husband’s alcohol drinking [21], wives’ lower economic status than husbands’, and women’s unemployment [22]. Previous studies in India reported health problems associated with IPV among women including poor mental health including mental disorders, depressive disorders, attempted suicide [23–26], terminated pregnancy [27], gynecological complaints, low Body Mass Index, and sexually transmitted infections [28].

The purposes of this study are to describe the lifetime prevalence of IPV, and to examine the association between IPV and health and well-being among women utilizing community health services for the economically disadvantaged in India. This study focuses on spousal abuse as IPV because marriage is related to social and cultural pressures that control the position of women in family and society in India [29]. The primary aim of the community organization where this study was performed is to promote community health. The organization was founded about 70 years ago and has more than 3,000 member institutions throughout India. The services provided at the organization and member institutions include prevention and treatment of communicable and non-communicable diseases, advocacy to promote “health as a right for all”, disaster management, and disability rehabilitation. There are two main reasons that this study targeted women utilizing community health services for the economically disadvantaged in particular. First, while women utilizing such community health services lack access to regular healthcare and may be at risk of poor health and IPV, little information is available about their abuse experience and health. Second, the knowledge about what community health organizations may offer to prevent IPV to ensure women’s health and safety in India is lacking. This study contributes to expanding the literature on health and abuse experience and to providing knowledge for developing intervention programs and research projects to improve the health and safety of women.

## Methods

### Study design and participants

The current project was a cross-sectional facility based study and based on the collaboration between a nonprofit community health organization in India and a research team of an academic institution in the US. The staff of the community organization and the research team worked together to develop the survey instrument, study protocol, participant recruitment strategies, and interpreting study results. The data collection was performed in Rajkot city in Gujarat, India. The state of Gujarat is located on the north-west coast of India. Rajkot city is in the center of the state with a population of approximately 1.3 million.

This study was approved by the University of Utah Institutional Review Board (IRB). The community organization provided a letter to permit data collection to the IRB. To ensure participants’ anonymity, identifiable personal information (e.g. name, phone number, address) was not collected. All survey materials including a consent cover letter, a flyer and a survey instrument, were available to participants in Gujarati (a main language in the state of Gujarat). A native Gujarati speaker, who is fluent in English, translated English materials into Gujarati. Another native Gujarati speaker, who is fluent in English, conducted the back-translation for the survey instrument. The study team then checked the accuracy of the translation. When the back-translation had a different meaning from the original English version, the forward- and the back-translations were re-done. This process was repeated three times. A third bilingual speaker checked translation accuracy in the case of differing translations.

The data were collected at 18 community health centers that are primarily for the economically disadvantaged (in 14 slums of Rajkot city and four villages within Rajkot district) in Gujarat, India for 47 days in the fall of 2013. The study included all community health centers of the community organization in Rajkot, Gujarat. The community health centers ask clients to pay only a minimum fee. Although the community health centers provide general health care and serve women and men, the majority of people who utilize services at the centers are women.

Study samples were convenience samples and were women who were aged 18 years or older, spoke and read Gujarati, and were seeking services at a community health center. Literacy of a potential participant was assessed based on a self-report. The staff who took main responsibility of the data collection reported that approximately 65-70% of the health centers’ patients were literate during the time of the data collection. The potential participants were visiting the community health centers primarily for seasonal illness. Out of the 219 participants who completed the survey, 169 participants had ever been married (currently married, widowed, or divorced). Among ever-married participants, 83 women had experienced IPV while 84 women had never experienced IPV. Two of the ever-married participants did not indicate whether they had been abused by their spouse or not. After excluding the two participants, 167 participants were included in analysis.

### Data collection

The 11 staff members of the community health centers collected data, on average three hours per day. The staff members received training on the data collection procedure and informed consent at the community organization in Gujarat. Recruitment occurred at a community health center by distributing flyers explaining the survey to clients in the waiting room. If a potential participant expressed interest in participating in the study, she received a consent cover letter and a self-administered paper survey. The staff members were available to answer questions from participants before consenting to participation and during the survey participation.

### Measures

#### Demographic characteristics

Demographic questions included age, educational level, current employment status and sector (government or private), marital status, household income per month in Indian Rupees, number of people living in the household, religion, caste, number of children, type of house (a Pakka house which is solid made of concrete, stone, brick or cement, or a Kuccha house made of mud or hay stack or tin roof), and living area (urban or rural).

#### Perceived health status

Perceived health status was measured using the Duke Health Profile (DUKE). The DUKE consists of 17 items and includes health measures (physical, mental, social, general, perceived health, and self-esteem), and dysfunction measures (anxiety, depression, pain, and disability) [30]. About half of the measures assessed health status over the past week while the remaining examined the current condition. Social health refers to participation in social activities and relationships with family or other people [31]. The DUKE uses a 3-point Likert scale except for the last question asking the days of “Stay in your home, a nursing home, or hospital because of sickness, injury, or other health problem” using None/1-4 days/5-7 days. Scoring (the score range 0–100) was based on the user manual [31]. Higher scores indicate better health status for the health measures and worse status for dysfunction measures. There is no specific cut-offs for health and dysfunction and the scores are considered continuous. The validity and reliability of the DUKE has been tested [31]. The DUKE has been translated into more than 17 languages and has been widely used in and outside of the US [31] including in India [32].

#### Depression

The Patient Health Questionnaire (PHQ)-9 measures levels of depression and asks how often a participant has been bothered by nine kinds of problems in the past two weeks such as *little interest or pleasure in doing things, feeling tired or having little energy,* and *poor appetite or overeating* using a 4-point Likert scale (0 = not at all and 3 = nearly every day). The PHQ-9 scores are total scores of the nine items (total score range 0–27). Depression severity is defined as: minimal, 0–4; mild, 5–9; moderate, 10–14; moderately severe, 15–19; severe, 20-27 [33]. The PHQ-9 scores were used for describing the overall level of self-reported depression. The responses were not verified by a clinician. The PHQ-9 is a valid and reliable tool and has been widely used internationally including in India [34–36]. While the DUKE has a depression measure, an additional depression measure from the PHQ-9 was added because depression is an important outcome. Previous studies showed the significant association between IPV and depression [25] and high prevalence of depression in slum communities related to IPV [37] in India.

#### Somatic symptoms

The Patient Health Questionnaire (PHQ)-15 is a valid, 15-item measure of somatic symptoms [38]. The PHQ-15 asks respondents to report somatic symptoms that they have experienced in the past four weeks using a 3-point Likert scale (0 = Not bothered at all, 1 = Bothered a little, 2 = Bothered a lot; with total score ranging from 0–30). Examples of somatic complaints represented by the items include stomach pain, back pain, and headaches. The PHQ-15 scores are the total scores from the 15 items and are defined as: no somatic disorder 1–4; mild somatization disorder 5–9; moderate somatization disorder 10–14; severe somatization disorder 15+. The PHQ-15 is a valid and reliable measure of somatic symptoms and has been used in various countries [39–42] including in India [43, 44]. But the information of the validity and reliability of the PHQ-15 in India or Gujarat is not available.

#### IPV experience

IPV experience was measured using questions extracted from a research project on IPV and help-seeking among Asian Indian, Pakistani and Filipino immigrant women in the US with permission from the primary author of the questionnaire [45]. The study team used the questions because those questions are comprehensive, have been used for Asian Indian women, and fit the social context of Indian society. The participants who had been married but were not currently married answered based on her ex- or late- husband. The first question asked the nature of marriage (e.g. love marriage, arranged marriage, forced marriage). Then, the participants were asked if their husband physically abused (e.g. pushed, grabbed, hit, slapped, kicked, used knife), sexually abused (e.g. forced or attempted to force sex), or emotionally abused them (e.g. denied access to money, made unwanted phone calls, separated or took away children) at least once sometime in their life. Participants answered whether they did or did not experience each kind of incident. The scoring was based on lifetime prevalence for each type of abuse (physical abuse, sexual abuse, or emotional abuse) (1 = experienced in lifetime, 0 = never experienced). Finally, the participants were asked to whom they sought help for abuse from husband (e.g. did not seek help, parents, health care facilities; 1 = yes or 0 = no) for each item.

### Data analysis

Data were analyzed using SPSS (version 19). Descriptive statistics were used to describe the distribution of the outcome and independent variables. Descriptive data were presented as means with standard deviations (SDs) for continuous variables, and frequencies and percentages for categorical variables. The ever-married participants were classified into two groups: participants who had ever experienced any type of IPV; and those who had never experienced IPV. These two groups were compared using Pearson Chi-square for categorical variables and independent samples t-test for continuous variables. Logistic regression was performed to examine the association between socio-demographic characteristics (i.e., age, education, work status, length of marriage, number of children, monthly income per person, caste, type of house, living area) and the lifetime experience of IPV.

Multiple linear regression analysis was conducted to test the association between health and dysfunction, and IPV and socio-demographic characteristics among ever-married participants. Each health or dysfunction measure was examined using separate models. Regression coefficients (standard errors) were used to obtain a 95% confidence interval.

## Results

Table 1 describes the socio-demographic characteristics of the 167 participants. The average age of the participants was 35.2 years (SD = 9.2). More than one third of the participants (n = 59, 35.3%) had secondary education (eighth grade) or higher level of education. Approximately 60% of the participants (n = 104) were employed (not including domestic work or seasonal farm work). The majority of the participants (n = 160, 95.8%) were currently married. The average length of marriage was 13.7 years (SD = 8.6). The average monthly income per person (monthly household income divided by the number of people living in the household) was 2,930.0 Indian Rupees (SD = 4,007.7) or 46.9 US Dollars (1 Indian Rupee = 0.016 US Dollar, December 2013; Average monthly income per person in Gujarat in 2011–2012 was 1,430.1 in rural areas and 2,472.5 in urban areas in Indian Rupee) [46]. The average number of people in the same household was 5.1 (SD = 1.6). More than ninety percent of the participants (n = 153) reported their religious belief was Hindu. One fourth of the participants (n = 42, 25.1%) reported their caste was general (highest). More than half of the participants (n = 92, 55.1%) lived with in-laws (husband’s parents). The average number of children was 1.9 (SD = 1.1). While approximately 70% of the participants (n = 115, 68.9%) lived in a Pakka house, solidly made of concrete, stone, brick or cement, others (n = 45, 26.9%) lived in a Kuccha house made of mud or hay with a tin roof. One third of the participants (n = 58, 34.7%) lived in an urban area. Arranged marriage was common among the participants (n = 142, 85%). There was no significant demographic difference between ever-married participants with IPV experience and those without IPV experience.

**Table 1:** **Participant socio-demographic characteristics**

	Total (N = 167)	Ever-married with IPV (n = 83)	Ever-married without IPV (n = 84)	p-value ^+^
Mean age, years	35.2 (9.2)	34.8 (8.3)	35.9 (10.1)	N.S.
Education				N.S.*
Less than primary (5^th^ grade)	28 (16.8)	12 (14.5)	16 (19.0)	
Completed primary (5^th^ grade)	22 (13.2)	11 (13.3)	11 (13.1)	
Less than upper primary (7^th^ grade)	6 (3.6)	4 (4.8)	2 (2.4)	
Completed upper primary (7^th^ grade)	44 (3.6)	24 (28.9)	20 (23.8)	
Secondary (8-10^th^ grade)	40 (24.0)	17 (20.5)	23 (27.4)	
Higher secondary (11-12^th^ grade)	15 (9.0)	8 (9.6)	7 (8.3)	
College education	2 (1.2)	2 (2.4)	1 (1.2)	
Graduate education	2 (1.2)	2 (2.4)	0	
Currently employed	104 (62.3)	54 (65.1)	50 (59.5)	N.S.
Employment sector				
Government	8 (4.8)	4 (4.8)	4 (4.8)	
Private	35 (21.0)	19 (22.9)	16 (19.0)	
Marital status				
Married	160 (95.8)	81 (97.6)	79 (94.0)	
Widowed	6 (3.6)	1 (1.2)	5 (6.5)	
Divorced	1 (0.6)	1 (1.2)	0	
Length of marriage	13.7 (8.6)	13.6 (7.3)	13.8 (9.8)	N.S.
Income per person/month in Rupee	2930.0 (4007.7)	3043.7 (4080.1)	2800.0 (3948.7)	N.S.
Number of people living in the household	5.1 (1.6)	5.1 (1.7)	5.1 (1.4)	N.S.
Religious belief				
Hindu	153 (91.6)	73 (88.0)	80 (95.2)	
Muslim	10 (6.0)	7 (8.4)	3 (3.6)	
Christian	2 (1.2)	2 (2.4)	0	
Caste				
General (highest)	42 (25.1)	19 (22.9)	23 (27.4)	N.S.
Other Backward Castes	51 (30.5)	28 (33.7)	23 (27.4)	
Schedule Caste/Schedule Tribe (lowest)	62 (37.1)	30 (36.1)	32 (38.1)	
Living with in-laws (husband’s parents)	92 (55.1)	44 (53.0)	46 (54.8)	N.S.
Number of children	1.9 (1.1)	1.9 (1.3)	1.8 (1.0)	N.S.
Type of house				
Pakka (concrete, stone, brick, cement)	115 (68.9)	57 (68.7)	58 (69.0)	N.S.
Kuccha (mud or hay stack or tin roof)	45 (26.9)	22 (26.5)	23 (27.4)	
Living area				
Urban	58 (34.7)	33 (39.8)	25 (29.8)	N.S.
Rural	106 (63.5)	49 (59.0)	57 (67.9)	
Type of marriage (if a participant had been married)				
Love marriage	6 (3.6)	4 (4.8)	2 (2.4)	
Arranged by parents and other relatives	142 (85.0)	64 (77.1)	78 (92.9)	
Forced by parents and other relatives	13 (7.8)	12 (14.5)	1 (1.2)	
Through internet	2 (1.2)	1 (1.2)	1 (1.2)	

*Note*. No. (%) or Mean (*SD*).

*Compared based on whether the participant completed secondary education or higher.

^**+**^ *p*-value denotes significant Chi-Square tests between categorical variables, and independent samples t-tests for continuous variables, comparing between ever-married participants with IPV experience and those without IPV experience.

Table 2 summarizes IPV experience and help-seeking for spousal IPV among participants who had experienced IPV. Logistic regression was performed to examine the impact of socio-demographic characteristics on the lifetime experience of IPV (not shown on the table). None of the socio-economic factors were associated with IPV. Nearly 40% of the participants with IPV (n = 31, 37.3%) experienced physical IPV only. Although physical abuse only was most common, many women experienced multiple forms of violence. Nearly 30% of the ever married women with IPV (n = 23, 27.7%) experienced all three forms of IPV. Other types of abuse included physical and emotional IPV (n = 12, 14.5%), physical and sexual IPV (n = 8, 9.6%), emotional IPV only (n = 8, 9.6%), and sexual and emotional IPV (n = 1, 1.2%). The most common physical IPV incident was “pushed, grabbed, or shoved” (n = 50, 29.9%) followed by “kicked” (n = 36, 21.6%). Husbands attempting or having actually forced the participant to have sex against her will was common sexual IPV (attempted n = 28, 16.8%; actually forced n = 24, 14.4%). “Damaged property” was the most common emotional IPV (n = 32, 19.2%) followed by “separated or took away children against your wishes” and “isolated, restricted, or controlled; did not give you enough food, clothing, medical care, etc.” (n = 29, 17.4% for each item). Approximately 45% of the participants with IPV experience (n = 37) did not seek any help for IPV. Seeking help from informal sources (e.g. parents 47%, siblings 18.1%, friends or neighbors 14.5%) was more common than that from formal help sources (e.g. social service organization 12%, health care facilities 8.4%, police or law enforcement 8.4%).

**Table 2:** **IPV experience and help-seeking**

	Ever-married with IPV (n = 83)
Spousal IPV	
Emotional abuse only	8 (9.6)
Physical abuse only	31 (37.3)
Physical & emotional abuse	12 (14.5)
Sexual & emotional abuse	1 (1.2)
Physical & sexual abuse	8 (9.6)
Physical, sexual & emotional abuse	23 (27.7)
Physical IPV incidents (multiple answers)	
Pushed, grabbed, or shoved	50 (29.9)
Kicked	36 (21.6)
Hit, slapped, or punched	32 (19.2)
Strangled or choked	12 (7.2)
Used knife, gun or other object (bat, bleach/acid)	9 (5.4)
Sexual IPV incidents (multiple answers)	
Attempted to force you to have sex against your will	28 (16.8)
Forced you to have sex against your will	24 (14.4)
Forced you to have sex with others	13 (7.8)
Emotional IPV (multiple answers)	
Damaged property	32 (19.2)
Separated or took away children against your wishes	29 (17.4)
Isolated, restricted, or controlled you; did not give you enough food, clothing, medical care, etc.	29 (17.4)
Denied access to money, jewelry, or other personal possessions	27 (16.2)
Followed, spied on, stood outside home/work, or had someone else do that	21 (12.6)
Made unwanted phone calls, text-messages, left unwanted letters, emails, gifts or items, or had someone else do that	14 (8.4)
Help-seeking for IPV (multiple answers)	
Did not seek help	37 (44.6)
Parents	39 (47.0)
Siblings	15 (18.1)
Child	7 (8.4)
Other family	10 (12.0)
In-laws	5 (6.0)
Friends or neighbors	12 (14.5)
Health care facilities	7 (8.4)
Social service organizations	10 (12.0)
Police/ law enforcement	7 (8.4)
Other	2 (2.4)

Frequency (%).

Table 3 presents physical and mental health comparisons between ever-married participants with IPV experience and those without IPV experience. Ever-married participants with IPV experience reported poorer physical health (p < 0.05), mental health (p < 0.01), and social health (p < 0.05), and lower self-esteem (p < 0.01) compared to ever-married participants without IPV experience. In addition, ever-married participants with IPV experience reported higher levels of anxiety (p < 0.01), higher levels of depression (DUKE-depression and PHQ-9, p < 0.01) and more somatic symptoms (p < 0.05) than those without IPV experience. While ever-married participants with IPV experience reported mild depression (PHQ-9 score = 5.7, SD = 7.6) and mild somatization disorder (PHQ-15 score = 6.5, SD = 8.8), those without IPV experience reported minimal depression (PHQ-9 score = 2.5, SD = 3.7) and no somatic symptoms (PHQ-15 score = 3.8, SD = 5.2).

**Table 3:** **Physical and mental health**

	Total (N = 167)	Ever-married with IPV (n = 83)	Ever-married without IPV (n = 84)	p-value ^+^
DUKE-health^a^				
Physical health	72.4 (29.2)	67.7 (31.3)	77.0 (26.4)	<0.05
Mental health	63.9 (18.2)	59.3 (16.7)	68.3 (18.6)	<0.01
Social health	49.7 (20.8)	45.6 (20.6)	53.8 (20.3)	<0.05
Perceived health	79.9 (34.0)	79.0 (34.3)	80.7 (33.9)	N.S.
Self-esteem	63.5 (16.6)	59.3 (14.0)	67.2 (17.9)	<0.01
DUKE-dysfunction^b^				
Anxiety	36.0 (18.7)	41.4 (17.2)	30.9 (18.7)	<0.01
Depression	38.6 (20.8)	43.9 (17.2)	33.7 (22.7)	<0.01
Pain	29.8 (36.1)	33.7 (37.5)	25.9 (34.4)	N.S.
Disability	3.9 (16.6)	5.5 (19.3)	2.4 (13.3)	N.S.
PHQ-9 (depression)^c^	4.1 (6.2)	5.7 (7.6)	2.5 (3.7)	<0.01
PHQ-15 (somatic symptoms)^d^	5.1 (7.3)	6.5 (8.8)	3.8 (5.2)	<0.05

Mean (SD).

^a^Higher score indicates better health status.

^b^Higher score indicates worse health status.

^c^Higher score indicates a higher level of depression.

^d^Higher score indicates more somatic symptoms.

^+^Independent samples t-tests comparing between ever-married participants with IPV and those without IPV.

Table 4 summarizes predictors of physical and mental health (physical health, mental health, social health, and self-esteem from the DUKE) among ever-married participants. Perceived health was not included because there was no significant variation among the participants. Spousal IPV was associated with poorer physical health (*β* = -15.2; p < 0.01), mental health (*β* = -12.82; p < 0.01), and social health (*β* = -9.35; p < 0.05), and lower levels of self-esteem (*β* = -10.97; p < 0.01). Being in the highest caste (general) was associated with better social health (*β* = 10.52; p < 0.05).

**Table 4:** **Predictors of physical and mental health among ever-married participants**

Dependent variables	Physical health ^a^	p-value	Mental health ^a^	p-value	Social health ^a^	p-value	Self-esteem ^a^	p-value
	***β***		***β***		***β***		***β***	
Independent variables								
IPV	-15.20	<0.01	-12.82	<0.01	-9.35	<0.05	-10.97	<0.01
Age	-0.20	N.S.	0.27	N.S.	0.60	N.S.	0.30	N.S.
Education – secondary or higher	3.53	N.S.	-6.00	N.S.	0.25	N.S.	0.70	N.S.
Employed	2.70	N.S.	0.19	N.S.	3.55	N.S.	3.38	N.S.
Length of marriage	-0.23	N.S.	-0.27	N.S.	-0.10	N.S.	0.23	N.S.
Number of children	1.03	N.S.	-1.33	N.S.	-3.00	N.S.	-2.52	N.S.
Income per person	0.001	N.S.	0.000	N.S.	-0.001	N.S.	0.000	N.S.
Caste – general (highest)	11.0	N.S.	4.94	N.S.	10.52	<0.05	7.14	N.S.
Living in Pakka house	0.15	N.S.	3.22	N.S.	-2.38	N.S.	4.50	N.S.
Living in urban area	9.33	N.S.	2.09	N.S.	7.04	N.S.	5.65	N.S.
*R* ^2^	0.15		0.16		0.17		0.20	
*F*	2.02		2.01		2.17		2.59	
*P*-value	<0.05		<0.05		<0.05		<0.05	

N = 167.

^a^Higher score indicates better health.

p-values denote significance from multivariate regression analyses.

Table 5 describes predictors of dysfunction (anxiety, depression and pain from the DUKE), depression (from the PHQ-9) and somatic symptoms (from the PHQ-15) among ever-married participants. Disability from the DUKE was not included because there was no significant variation among the participants, few of whom reported disability. Spousal IPV was associated with higher levels of anxiety (*β* = 16.79; p < 0.01), depression (DUKE depression, *β* = 16.19, p < 0.01; PHQ-9, *β* = 4, p < 0.01), and pain (*β* = 12.78, p < 0.05), and more somatic symptoms (*β* = 3.46, p < 0.05). Being in the highest caste was associated with lower levels of anxiety (*β* = -1.00, p < 0.05) and pain (*β* = -16.10, p < 0.05) and fewer somatic symptoms (*β* = -3.53. p < 0.05). Higher educational level was associated with higher levels of depression (*β* = 9.20, p < 0.05) from the DUKE depression measure. There was no such effect from the PHQ-9 depression measure, however.

**Table 5:** **Predictors of dysfunction, depression and somatic symptoms among ever-married participants**

Dependent variables	Anxiety ^a^	p-value	Pain ^a^	p-value	DUKE depression ^a^	p-value	PHQ-9 depression ^b^	p-value	PHQ-15 somatic symptoms ^c^	p-value
	***β***		***β***		***β***		***β***		***β***	
Independent variables										
IPV	16.79	<0.01	12.78	<0.05	16.19	<0.01	4.00	<0.01	3.46	<0.05
Age	-0.52	N.S.	0.67	N.S.	-0.47	N.S.	0.15	N.S.	0.08	N.S.
Education – secondary or higher	4.24	N.S.	-8.12	N.S.	9.20	<0.05	-0.58	N.S.	0.37	N.S.
Employed	-0.83	N.S.	-4.58	N.S.	0.003	N.S.	-1.78	N.S.	-1.77	N.S.
Length of marriage	0.62	N.S.	-0.24	N.S.	0.74	N.S.	-0.10	N.S.	0.08	N.S.
Number of children	0.21	N.S.	-1.04	N.S.	0.56	N.S.	0.16	N.S.	-0.50	N.S.
Income per person	0.000	N.S.	-0.001	N.S.	0.000	N.S.	0.000	N.S.	0.000	N.S.
Caste – general (highest)	-1.00	<0.05	-16.10	<0.05	-1.72	N.S.	-2.55	N.S.	-3.53	<0.05
Living in Pakka house	-5.63	N.S.	8.80	N.S.	-3.81	N.S.	-1.21	N.S.	0.59	N.S.
Living in urban area	-4.76	N.S.	-9.84	N.S.	-3.54	N.S.	-1.18	N.S.	-2.05	N.S.
*R* ^2^	0.27		0.19		0.20		0.17		0.14	
*F*	3.93		2.99		2.70		2.59		2.03	
*P*-value	<0.01		<0.01		<0.01		<0.01		<0.05	

N = 167.

^a^Higher score indicates higher levels of dysfunction.

^b^Higher score indicates a higher level of depression.

^c^Higher score indicates more somatic symptoms.

p-values denote significance from multivariate regression analyses.

## Discussion

This study described the lifetime prevalence of IPV, and examined the association between IPV and physical and mental health well-being among women utilizing community health services for the economically disadvantaged in India. There were three main findings in this study. First, participants who had experienced IPV reported poorer physical and mental health compared to those who had not. Second, many women experienced multiple types of IPV. Third, while being in the highest caste was a significant factor associated with better health, caste and other socio-demographic factors were not associated with IPV.

Participants who had experienced IPV reported poorer physical and mental health than those without IPV. In particular, IPV experience was associated with anxiety, pain, depression, somatic symptoms as well as lower levels of social health and self-esteem. The results are consistent with previous studies [1–3, 10]. Women who experience IPV suffer from a wide range of health problems. Providing IPV prevention and intervention would be important to improve women’s health. Nevertheless, the majority of the participants with IPV experience either did not seek any help or relied on informal help sources. The results on help seeking behaviors were consistent with a previous study in India: only one third of women sought help for the last IPV incident and the majority of them sought help from informal sources mainly from parents [47]. Only 8.4% of participants with IPV experience in the current study sought help for IPV from healthcare facilities. Barriers to seeking help need to be identified and eliminated to ensure health and safety of women experiencing IPV.

While physical abuse only was the most common IPV experience, more than half of the participants with an IPV history experienced all types of or two types of IPV. There is a study from New Zealand which examined overlap of types of IPV [48]. The results of the New Zealand study indicated that the most common abuse was psychological abuse only. Similarly, a study from Japan shows that emotional IPV was more commonly experienced than physical and sexual IPV among women with an IPV history [12]. However, the results of the current study suggest that physical IPV (physical IPV only or physical IPV plus sexual and/or emotional IPV) is the most common type of IPV in India. While this study was not able to assess physical and mental health status by type of violence experience due to small sample size, evidence is mixed on this issue and additional research is needed. Future research would warrant how the relatively high percentage of physical IPV and emotional abuse in India would affect the health of women who had experienced IPV differently from other countries.

It may be challenging for abused women to seek help particularly for IPV because of fears to disclose abuse or lack of awareness of available formal resources [47]. The women in the current study were seeking community health services for seasonal illness. If community health centers provide prevention and intervention for IPV along with their regular health services, it may be easier for abused women to seek help from health care facilities for IPV. IPV screening at a facility would be another potential intervention, though a number of challenges have been reported about implementing an IPV screening program, such as lack of time and organizational support [49] and also the limitations of IPV screening programs (e.g. low identification rate of IPV, insufficient evidence of the effectiveness of IPV screening to improve women’s health) [50]. In addition to facility-based interventions, community-level interventions would be necessary as community norms are significantly associated with spousal physical IPV in India [51]. A community based IPV prevention program using campaign activities which have been implemented in the Gujarat community in the US [52] may be one of the models for community health centers in Gujarat to include IPV prevention and intervention in their regular services. The program in the Gujarat community in the US distributes IPV prevention messages through social marketing and incorporates community engagement for developing campaign strategies and implementations. Further research and practice are necessary to develop IPV prevention and intervention programs which fit community health centers in India.

Besides IPV, the most influential factor on health was caste. Participants in the highest caste (general) reported better health than those in the lower caste. Previous studies show that caste was associated with the utilization of and access to maternal and reproductive health care [53, 54], childhood immunization [55], the receipt of state financial support [56], and household health expenditure [57]. In any case, caste is not something that individuals can change. Any health-related barriers that people in lower castes face should be eliminated to ensure rights to healthcare for everyone.

Contrary to previous studies [21, 22], there was no significant difference in socio-demographic characteristics such as caste, low household income, low education levels, and women’s work status between participants with IPV experience and those without IPV experience. There are two possible explanations of the results. First, it is possible that there were few variations in socio-demographic characteristics among participants because all of the participants were seeking health care at a community health center for the underserved. Second, husbands’ socio-demographic characteristics (e.g. husband’s educational level, occupation, use of alcohol) often influencing IPV occurrence [58, 59] were not included in the current study. Future studies should examine how socio-demographic characteristics of a husband and a wife would affect IPV and women’s health in India.

### Limitations

This study has some limitations. Because this study used a self-administrated survey, there were no illiterate participants. Women who do not have education have a higher prevalence of IPV compared to those with education [60]. Future research should include illiterate women using an interviewer-administrated survey. As this study was conducted among a facility-based sample of women, the participants of this study may be more likely to have lower mental and physical health scores than the general population. Although all scales used in this study are validated and reliable, the validity and reliability of these scales in India or in Gujarat is not available. This study was cross-sectional and could not examine causal relationships among the variables. The number of participants was too small to examine how types of IPV were associated with different physical or mental health issues. We did not have data on severity and frequency of IPV experience. Thus we were unable to classify the participants based on the severity of IPV which might have affected health differently. The participants of this study were seeking help at a community health center and therefore women in this study may also be more likely to demonstrate help-seeking behaviors than the general population. This might have affected the findings on the prevalence of help-seeking for IPV. Finally, the information about the characteristics of the participants’ husbands was not collected. Such information may be important because the characteristics of abusers are often related to IPV. For example, the educational level of male partners, not just that of women, affects the occurrence of IPV in India [61].

## Conclusions

The findings of this study contribute to a better understanding of IPV experience and perceived physical and mental health among women utilizing community health services in India. Women in India are at risk of IPV. Women with IPV experience are more likely to report poorer physical and mental health. Yet women experiencing IPV do not seek help or they rely on informal help sources. Community health organizations may take a role in IPV prevention and intervention because it would be easier for abused women to visit a community health center for general health issues and to receive additional services related to IPV if necessary rather than visiting a center particularly for IPV. Finally, implementing both facility-based and community-based interventions would be important to encourage more women with IPV experience to seek help.

## Authors’ information

AK is an Assistant Professor of Sociology at the University of Utah. She received PhD in Health Services Organizations and Policy and the Master of Social Work from the University of Michigan. Her current research is suited within two specific areas: global health research related to violence against women; and community-based research concerning healthcare for the underserved. VG is a Project Officer for the Catholic Health Association of India and is responsible for hospital consultation and children’s health programs. He also has experience in data management and analysis. KM is a doctoral student in the Department of Sociology at the University of Utah. She has worked at a Rape Crisis Center and a Domestic Violence shelter. TT is a Director General for the Catholic Health Association of India, which is one of the world’s largest non-governmental organizations in the health sector. He holds PhD in Social Work from the University of Utah and the Master of Social Work from the University of Mumbai.
